# A Numerical‐Experimental Assessment of the Dilute Phase and Erosion in a Larvae‐Killing Processing System: Considering the Geometry Variation

**DOI:** 10.1002/fsn3.70771

**Published:** 2025-08-03

**Authors:** Ali Ebadi, Adel Rezvanivand Fanaei, Ali Hassanpour, Vahid Rostampour

**Affiliations:** ^1^ Department of Mechanic of Biosystem Engineering Urmia University Urmia Iran

**Keywords:** control volume method, erosion rate, pressure drop, turbulent intensity

## Abstract

In the present study, the pneumatic conveying of wheat in the dilute phase focused on four different pipe cross‐section ratios including a/b = 1.5, a/b = 2, b/a = 1.5, and b/a = 2 has been experimentally and numerically examined. The system consists of the conveying pipe used inside the larvae system, which is used to transfer materials. Due to the Reynolds number calculations, the conveying is conducted in the turbulent regime. The combination of Reynolds Stress and Discrete phase models (RSM‐DPM) was used to model the fluid and solid phases, respectively. The dimensionless velocity magnitude, static and dynamic pressures, erosion, vorticity magnitude, and turbulence intensity contours were investigated in the four mentioned scenarios using ANSYS Fluent commercial software. According to the results, the inner radius of the elbows, and especially the first elbow, were the areas where the maximum velocity was observed in these sections. As a negative parameter, the maximum pressure drop was obtained with a value of 322 Pa at the cross‐section ratio of a/b = 2, which made the selection of this ratio a great challenge. Also, the maximum erosion rate occurred at the cross‐section ratio of a/b = 2, which is considered a negative parameter. Moreover, due to the rotational flows created in the inlet ratios b/a = 1.5 and b/a = 2, these ratios are not very practical in terms of application and will cause energy loss in the system through the interaction between the various flows. Finally, considering all scenarios among the four cross‐section ratios, the ratio of a/b = 1.5 was proposed as the most appropriate selection.

## Graphical abstract

The Reynolds stress (RSM) and Discrete phase models (DPM) were used to model the conveying of wheat in the dilute phase. The contours related to the velocity magnitude, static pressure, dynamic pressure, erosion, vorticity magnitude, and turbulence intensity were investigated in four different cross‐section ratios.

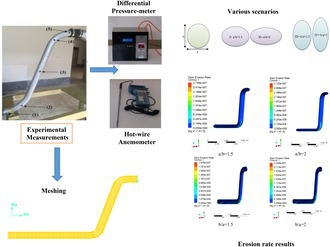

## 1 Introduction

The pneumatic conveying process of agricultural products offers significant potential for researchers. Several critical factors, including (1) the method of transportation, (2) the physical condition of the materials, and (3) flow characteristics, are crucial to achieving efficient conveying (Ghafori and Sharifi 2018). Optimizing the conveying paths, particularly in pipe elbows, can significantly decrease issues like reverse flow and blockages. In these elbows, wall‐particle collisions play a major role in increasing the pressure drop. As such, minimizing pressure drop through effective design optimization offers a substantial advantage (Mills 2004).

In engineering applications, optimization is often categorized into two primary areas: geometric optimization and operational optimization. Agricultural conveying via pipes is generally explored through experimental studies or numerical simulations. In experimental setups, measurements are conducted to obtain applicable data. However, with advancements in numerical methods and computational sciences, researchers can now gain deeper insights into internal flow behaviors. These applications include the evaluation of elbows, curved ducts, and bends, which induce secondary flows. Characterizing the secondary flow in a pipe bend downstream utilizing a computational study was carried out by various turbulence models (Kim et al. 2014). Also, a computational analysis of secondary flow inside a manifold bend was carried out to capture the velocity profile in bends (Banerjee et al. 2023). Similarly, other studies including the geometry effects of vertical (90°) elbow in air‐water multiphase flow; 3‐D numerical analysis of duct flow to in‐plate bends; and application of logarithmic law to obtain the velocity profile in a turbulent flow were carried out (Banerjee et al. 2024; Grossmann and Lohse 2017; Yadav et al. 2014).

Also, numerical simulations enable scientists to design highly accurate conveying systems at reduced costs and shorter times. In a recent study, momentous parameters like wall shear erosion rate and centrifugal force were investigated in a high efficiency Stairmand cyclone separator. (Dizajyekan et al. 2022; Naimi et al. 2019). Computational Fluid Dynamics (CFD) is one such advanced numerical approach used to analyze phenomena like fluid flow and heat transfer (Rezvanivand Fanaei et al. 2021). Additionally, modeling multiphase flows in conveying systems typically follows either the Eulerian or Lagrangian approach (Dizajyekan et al. 2021; Kuan et al. 2007; McGlinchey et al. 2007).

A study on pneumatic conveying of agricultural seeds highlighted two critical factors, including solid friction factor and pressure drop. The findings indicated that (1) pipe radius, (2) solid‐air ratio, and (3) air velocity significantly influence pressure drop (Raheman and Jindal 2001). Another study examined the impact of inlet velocity on damage to barley and corn during pneumatic conveying. The results showed that power consumption increased with enhancing air velocity, whereas pressure drop followed a nonlinear pattern. Velocity limits conducive to safe seed transport were determined as 20 m/s^−1^ for barley and 15 m/s^−1^ for corn (Ghafori et al. 2011).

An erosion‐based numerical‐experimental study was carried out to analyze the erosion in the sand conveying system elbow. In this study, erosion rate distribution is achieved using a conventional Eulerian–Lagrangian approach. It is found that surface roughness development, surface profile development, and particle shape had an effective effect on the erosion. As a conclusion, adopting an appropriate rough wall with the Eulerian–Lagrangian approach led to a more accurate prediction (Solnordal et al. 2015).

A heat exchanger including an interior pipe with fins and operated under counter‐flow conditions was studied numerically. Simulations conducted via ANSYS Fluent revealed that both effectiveness and Nusselt number improved with higher Reynolds numbers, while the friction factor decreased under the same conditions (Amalia et al. 2020). A new approach for calculating pressure drop in a conveying system was proposed by Shishkin et al. (2020). This method included additional terms aimed at reducing pressure losses by optimizing airflow. The experimental data validated their model with high accuracy (Shishkin et al. 2020).

Subsequent research explored CFD applications in agricultural fields, including energy optimization, separation processes, drying, and more (Akhoundzadeh Yamchi et al. 2024; AkhoundzadehYamchi et al. 2023; Albion et al. 2006; Fernández‐García et al. 2020; Gholamalizadeh and Kim 2014; Mohammadzadeh et al. 2022; Piri et al. 2021, 2024a, 2024b; Piscia et al. 2012; Zobeiri et al. 2021).

In a related numerical investigation, three elbow angles (105°, 120°, and 135°) in a larvae‐killing system were examined to identify optimal operating conditions. CFD simulations were performed using ANSYS Fluent software. The authors employed the RSM (Reynolds Stress Model) to simulate the fluid's continuous phase and the Discrete Phase Model (DPM) to represent particle behavior in the discrete phase. Findings concluded that the 120° elbow angle offered the best performance under operational conditions (Mohammadzadeh et al. 2021).

In another study, the anti‐erosion properties of various novel elbows in a conveying system were evaluated. Two structures contained triangular grooves and ribs, as well as square grooves and ribs were evaluated considering anti‐erosion resistance. Finally, the triangular single rib was selected as the best erosion resistance structure with a 47% increment at 20° (Guo et al. 2022).

In an innovative design, the effect of hemispherical protrusion on elbow erosion was studied. The optimization process could reduce the erosion rate by about 40%, which was achieved in a multi‐rows configuration. Moreover, the ability of dispersing in inhibiting the secondary flow, forming vortex and dispersing particle flow was determined (Li et al. 2022).

Xiao et al. (2023) carried out a numerical study about the investigation of wall erosion occurring within the elliptical chamber. The validation process was examined using the experimental pressure drop of a standard elbow. In this study, the particle size effect on erosion and flow characteristics was evaluated, and an elliptical geometry was proposed. In the proposed geometry, the erosion decreases by about 58% in comparison with the base elbow.

The larvae‐killing system is designed to delete larvae through physical collisions during material conveying. This system comprises components such as piping lines, elbows, feeders, blowers, and other elements. The piping section, consisting of straight pipes and elbows at specific initial lengths, plays a key role in determining critical parameters affecting the efficiency of material transport, such as pressure drop. Most research has concentrated on examining the elbow within conveying systems. However, the evaluation of varying cross‐section ratios in the larval killing system remains insufficiently explored, creating the impetus for this investigation. The primary innovations and objectives of this study can be outlined as follows:

- Conducting a thorough evaluation of the conveying process to validate simulation results with experimental data.
- Employing an advanced DPM to predict solid‐phase interactions within the conveying process.
- Analyzing and reporting key parameters such as dimensionless velocity magnitudes, static and dynamic pressures, erosion rates, turbulence intensity, and vorticity magnitudes across all scenarios.
- Using the surface‐particle Oka model to simulate erosion rates for each scenario.

## 2 Materials and Methods

### 2.1 Empirical Measurement System

**FIGURE 1:**
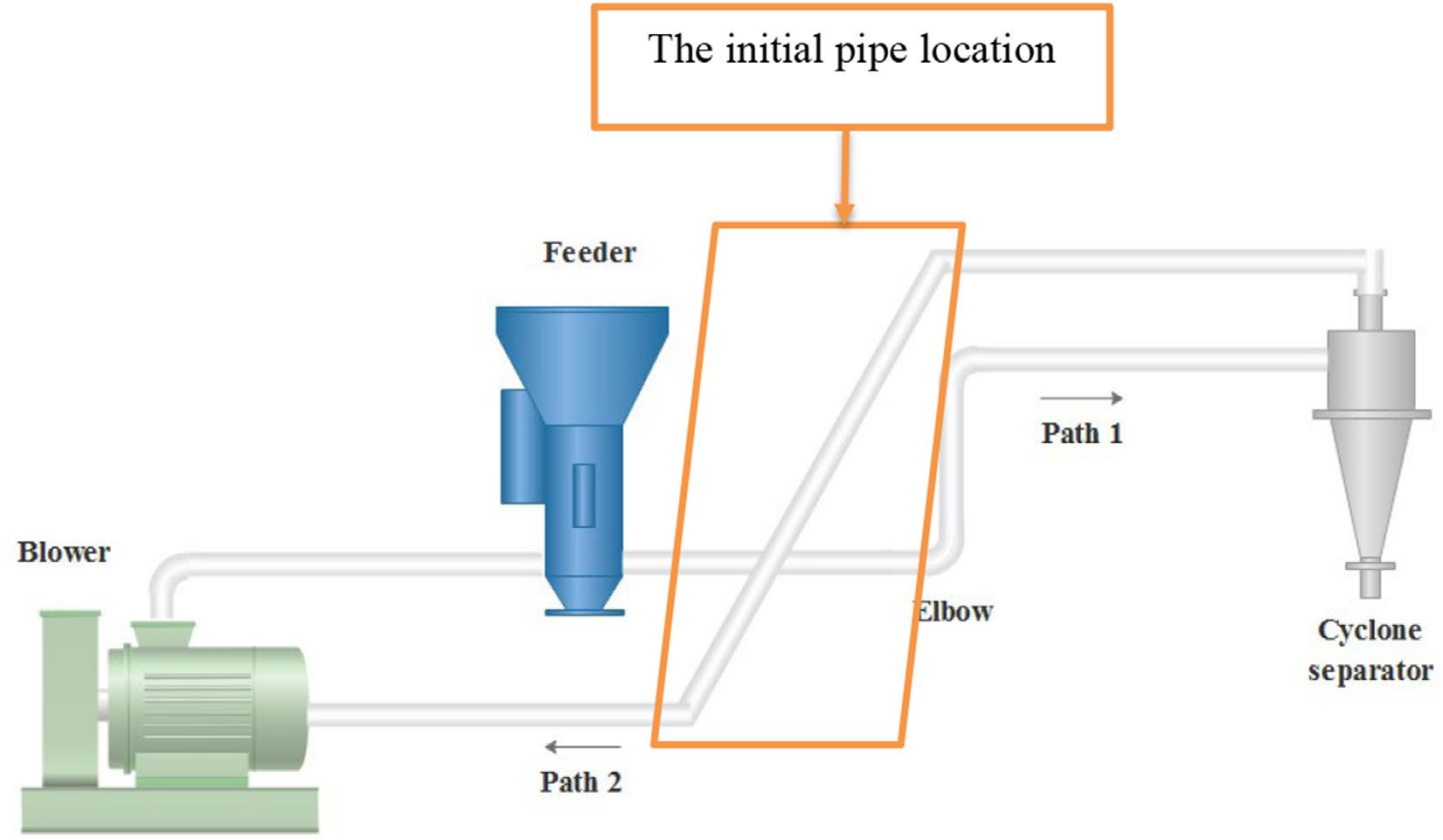
Conveying system.

**FIGURE 2:**
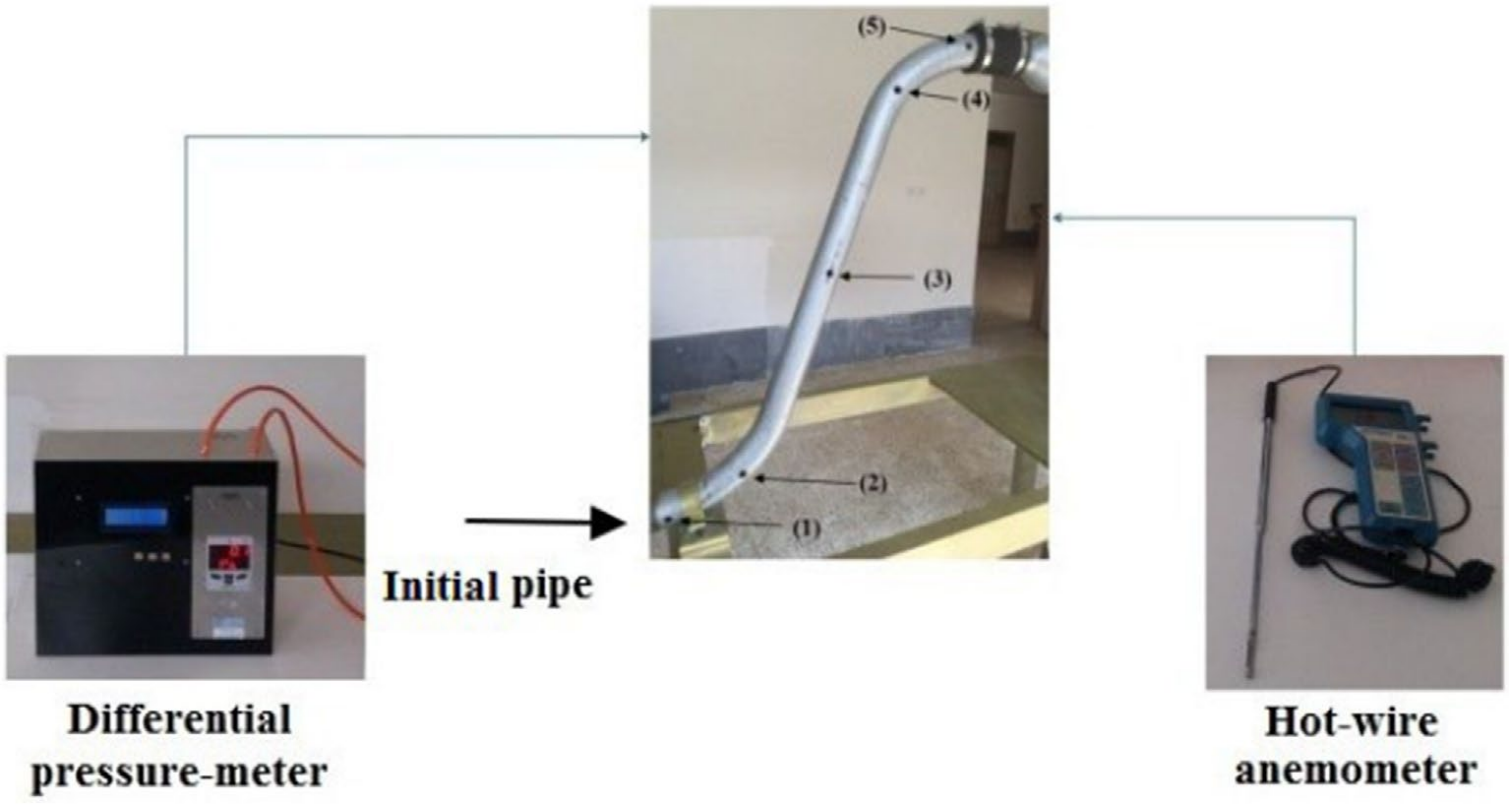
Instruments for measurement beside experimental setup.

A schematic representation of the empirical measurement system is shown in Figure 1. This system is applied in agricultural processes such as killing larvae. The ratio of cross‐sectional pipe emerges as a critical section in the conveying process. Figure 2 depicts the layout of the initial pipe alongside measurement points (points 1–5), where velocity data were collected using a hot‐wire anemometer for simulation boundary conditions. In addition, the pressure was measured using a pressure meter. The uncertainty, accuracy, and total error of the used instruments are summarized in Table 1.

**TABLE 1:** The uncertainty, accuracy, and total error of used instruments.

Instrument	Uncertainty	Accuracy	Total error
Hot‐wire anemometer	±0.1 m.s^−1^	0.1 m.s^−1^	0.18%
Differential pressure meter	±0.05 Pa	0.1 Pa	0.14%

### 2.2 New Design Scenarios

**FIGURE 3:**
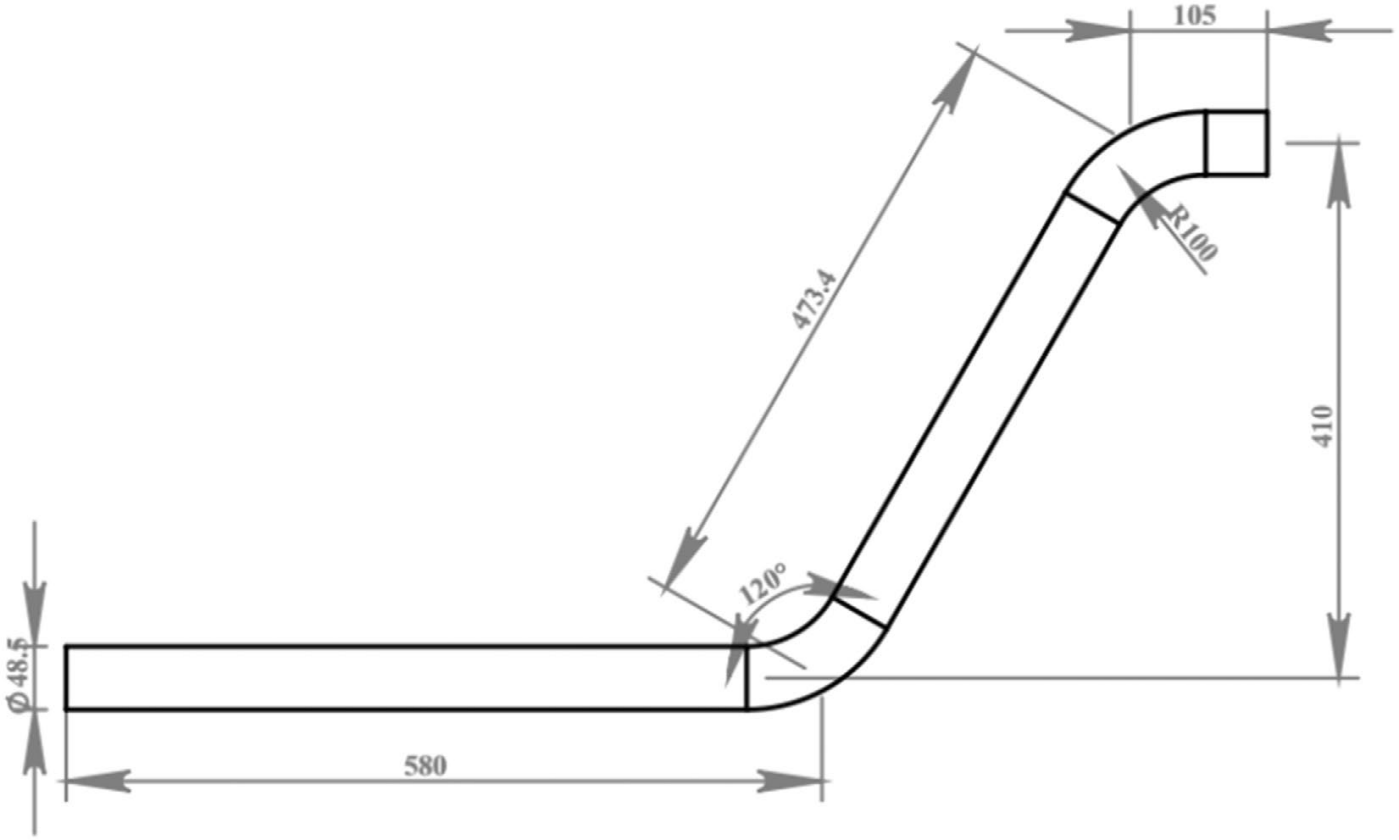
The dimensions of the experimental loop.

**FIGURE 4:**
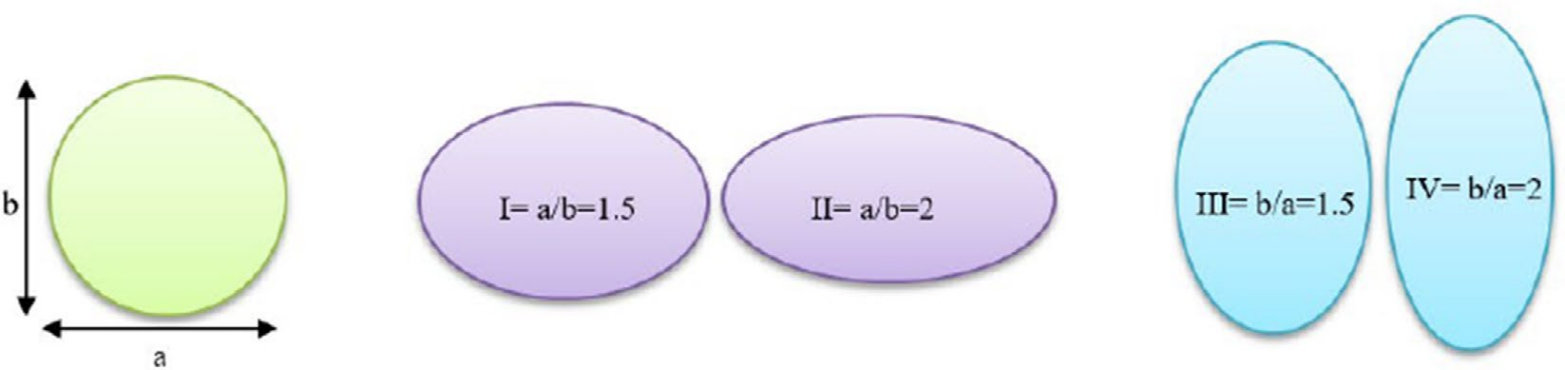
Four different scenarios as pipe cross‐section ratios.

Figure 3 illustrates a representation of the conveying pipe. The selection of 120° bend was based on a previous study conducted by Mohammadzadeh et al. (2021) which provided the best performance under operational conditions. The system is evaluated in four scenarios specified by cross‐section ratios as *a/b* = 1.5, *a/b* = 2, *b/a* = 1.5, and *b/a* = 2 (Figure 4). This component (cross‐section ratio) is crucial in facilitating and optimizing the flow dynamics. Actually, cross‐section ratios influence flow patterns, including secondary flows and vortex formation, which are crucial factors in understanding erosion behavior and pressure drops. The mentioned cross‐section ratios were chosen based on the feasibility of these ratios in terms of efficiency, manufacturing issues, and rational justification in their use.

### 2.3 Determine the Flow Regime and Selection of Solver

#### 2.3.1 Reynolds Number

In order to determine the flow regime, the Reynolds number of the fluid flow, which is a dimensionless number, was calculated by considering flow characteristics such as density, velocity, equivalent diameter, and dynamic viscosity. According to the values obtained for all four scenarios (Reynolds number above 4000), the fluid flow regime was identified as turbulent (Equation 1).

(1) Re=ρvdμ

where *ρ* is the density (kg.m^−3^), *v* is the velocity (m.s^−1^), *d* is the equivalent hydraulic diameter (m) and *μ* is the absolute or dynamic viscosity (Pa.s).

#### 2.3.2 Mach Number

The Mach number determines whether the flow is compressible or incompressible. Also, considering the fluid flow velocity and the sound speed in the medium, the Mach number was obtained, which, considering that it was below 0.3 in terms of value (Equation 2), was considered incompressible. It should also be noted that, considering the incompressibility of the flow, the pressure‐based solver was used for the computational solver.

(2) $$\mathrm{Ma}=\frac{v}{c}$$

where *v* is the local flow velocity (m.s^−1^) and *c* is the velocity of sound in the medium (m.s^−1^).

To determine the flow regime, the Reynolds number of the fluid flow was calculated by equation (1). According to the values obtained for all 4 scenarios (Reynolds number above 4000), the fluid flow regime was identified as turbulent.

Also, the dimensionless Mach number determines whether the flow is compressible or incompressible. Also, considering the equation (2), the Mach number was obtained below 0.3, which represents an incompressible flow. It should also be noted that due to the incompressibility of the flow, a pressure‐based solver was used for the computational solver.

### 2.4 Simulation Setup and Numerical Approach

The finite volume method, as implemented in ANSYS Fluent, was employed to solve the equations about laws of conservation (Rezvanivand Fanayi and Nikbakht 2015). In fact, selecting a proper turbulence model plays a critical role in achieving reliable results. While the k‐ε models offer significant computational efficiency; however, the RSM provides greater accuracy in capturing localized flow behaviors, particularly in regions experiencing intense erosion or near walls (Karbon and Sleiti 2020). Consequently, the RSM model was selected to model the turbulence in the present study. The equations governing the RSM model are outlined in equations ((3), (4), (5), (6), (7)).

(3) $$\frac{\partial }{\partial t}\left(\rho \;\bar{u_{i}^{\prime }u_{j}^{\prime }}\right)+\frac{\partial }{\partial x_{k}}\left(\rho u_{k}u_{i}^{\prime }u_{j}^{\prime }\right)=D_{\mathrm{ij}}+P_{\mathrm{ij}}+\prod \mathrm{ij}+\varepsilon_{\mathrm{ij}}+S$$

Where $u^{\prime }$ *and* $\bar{u}$ *are* dispersion and averaged velocities (m.s^−1^), and $\frac{\partial }{\partial x_{k}}\left(\rho u_{k}u_{i}^{\prime }u_{j}^{\prime }\right)$ is convective transport.

*D*_*ij*_ is the stress diffusion, which is defined by the equation (4):

(4) $$D_{\mathrm{ij}}=\frac{\partial }{\partial x_{k}}\left[\bar{\rho u_{i}^{\prime }u_{j}^{\prime }u_{k}^{\prime }}+\bar{\left(P^{\prime }u_{j}^{\prime }\right)}\delta_{\mathrm{ik}}+\bar{\left(P^{\prime }u_{i}^{\prime }\right)}\delta_{\mathrm{jk}}-\mu \left(\frac{\partial }{\partial x_{k}}\bar{u_{i}^{\prime }u_{j}^{\prime }}\right)\right]$$

*P*_*ij*_ is the shear production which is defined by the equation (5):

(5) $$P_{\mathrm{ij}}=-\rho \left[\bar{u_{i}^{\prime }u_{k}^{\prime }}\frac{\partial u_{j}}{\partial x_{k}}+\bar{u_{j}^{\prime }u_{k}^{\prime }}\frac{\partial u_{i}}{\partial x_{k}}\right]$$

∏ij is the pressure strain which defined by the equation (6):

(6) $$\prod \mathrm{ij}=p\left(\frac{\partial u_{i}^{\prime }}{\partial x_{j}}+\frac{\partial u_{j}^{\prime }}{\partial x_{i}}\right)$$

$\varepsilon_{\mathrm{ij}}$ is dissipation which defined by the equation (7):

(7) $$\varepsilon_{\mathrm{ij}}=-2\mu \frac{\partial {u_{i}}^{\prime }}{\partial {x_{k}}^{\prime }}\frac{\partial {u_{j}}^{\prime }}{\partial {x_{k}}^{\prime }}$$

And finally S is the source term.

### 2.5 Boundary Condition

Defining suitable boundary conditions is a crucial aspect of handling the mathematical formulations and ensuring alignment with the physical problem. The specific boundary conditions applied in the numerical calculations are summarized in Table 2.

**TABLE 2:** Boundary conditions.

Boundary conditions	Value/Condition
velocity inlet	20 m.s^−1^
Pressure outlet	Atmospheric pressure
Wall	No‐slip condition

Additionally, the Lagrangian multiphase model was utilized for simulating the solid phase in pneumatic conveying. The second phase characteristics incorporated into the Lagrangian multiphase model are detailed in Table 3.

**TABLE 3:** Second phase characteristics.

Property	Value
Solid density	770 kg.m^−3^
Average diameter	5^mm^

### 2.6 Discrete Phase Model

This model utilizes the Lagrangian approach to simulate particle‐fluid interactions. In this framework, momentum, mass, and energy exchanges occur between the fluid and dispersed phases. For the DPM, the volume fraction of the dispersed phase is restricted to less than 15% (Emami et al. 2024). The governing equations of particles in the Lagrangian approach are detailed in equations ((8), (9), (10)) (Aghaei et al. 2022).

(8) $$\frac{du_{\mathrm{pi}}}{\mathrm{dt}}=\frac{18\mu }{d_{p}^{2}\rho_{p}}C_{d}\frac{Re_{p}}{24}\left(u_{i}-u_{\mathrm{pi}}\right)+\frac{g_{i}\left(\rho_{p}-\rho \right)}{\rho_{p}}$$

(9) $$\frac{dX_{\mathrm{pi}}}{\mathrm{dt}}=u_{\mathrm{pi}}$$

(10) Rep=ρpdpu−upμ

where *ρ* is density (kg.m^−3^), *μ* is dynamic viscosity (Pas.s), *C*_*d*_ is the drag coefficient, *g*_*i*_ is gravitational acceleration, *u*_*i*_ is gas velocity (m.s^−1^), *u*_*pi*_ is particle velocity (m.s^−1^), *Re*_*p*_ is the relative Reynolds number, and *d*_*p*_ is particle diameter (m) (Elsayed and Lacor 2011).

### 2.7 Modeling of Erosion

Erosion is a key criterion in conveying, particularly concerning solid‐wall interaction. The procedure proposed by Oka and Yoshida (2005) is described as follows:

(11) $$\mathrm{ER}=1\times 10^{-9}\rho_{w}\mathrm{kF}\left(\theta \;\right)\left(H_{v}\right){\left(\frac{V_{p}}{V^{\prime }}\right)}^{k_{2}}{\left(\frac{d_{p}}{d^{\prime }}\right)}^{k_{2}}$$

(12) $$F\left(\theta \;\right)={\left(\sin\right)}^{\mathrm{nl}}{\left[1+H_{v}\left(1-\sin\theta \right)\right]}^{n2}$$

where $V^{\prime }$ is the reference velocity (m.s^−1^), *V*_*p*_ is the particle velocity (m.s^−1^), *H*_*v*_ is the Vickers number (Gpa) *d*_*p*_ and $d^{\prime }$ are the particle and reference diameters (m), respectively (m). The density and iron Vickers hardness is considered 7875 (kg.m^−3^) and 150 MPa, respectively. Also, *n1*, *n2*, $V^{\prime }$, *k*, *k*_*1*_, *k*_*2*_, *k*_*3*_, and $d^{\prime }$ are constants (Oka and Yoshida 2005).

### 2.8 Assumptions Used in Simulations

The air flow has a very low value in stagnation condition. Also, considering the ideal gas assumption will not have any significant errors in the calculations.

## 3 Results and Discussion

### 3.1 Mesh Independence Test

**FIGURE 5:**
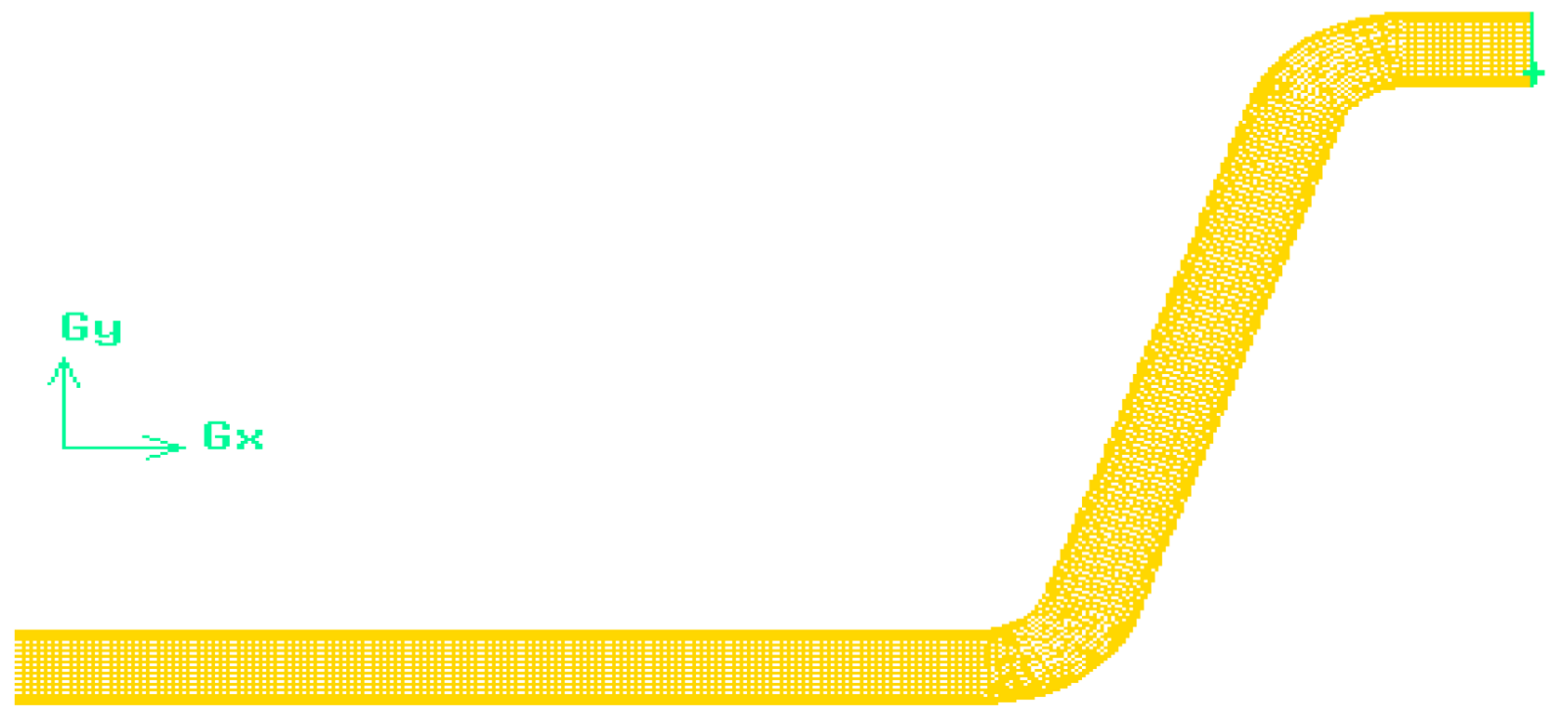
Meshing of the geometry.

The structured 3D meshes were utilized in the simulations (Figure 5). Also, the enhanced wall treatment is used for a more detailed simulation of phenomena like turbulence near walls. Moreover, the y + value as an index to determine the applicability of near‐wall turbulence models and the accuracy of capturing near‐wall flow behavior was 3. To improve calculation accuracy, fine meshes were applied in regions with high gradients. Four mesh configurations—labeled as I (164,658 numbers), II (278,325 numbers), III (492,112 numbers), and IV (907,204 numbers) were generated using Gambit software and subjected to a mesh independence tes. The test results indicated that the pressure drop difference between mesh levels III and IV was less than 5%, which is presented in Table 4. Consequently, considering both temporal efficiency and computational resources, the third level of mesh numbers was chosen for the simulations.

**TABLE 4:** Mesh independence test.

Mesh level	Mesh numbers	Pressure drop difference (%)
I	164,658	21.65
II	278,325	7.86
III	492,112	2.14
IV	907,204	1.95

### 3.2 Validation Process

**FIGURE 6:**
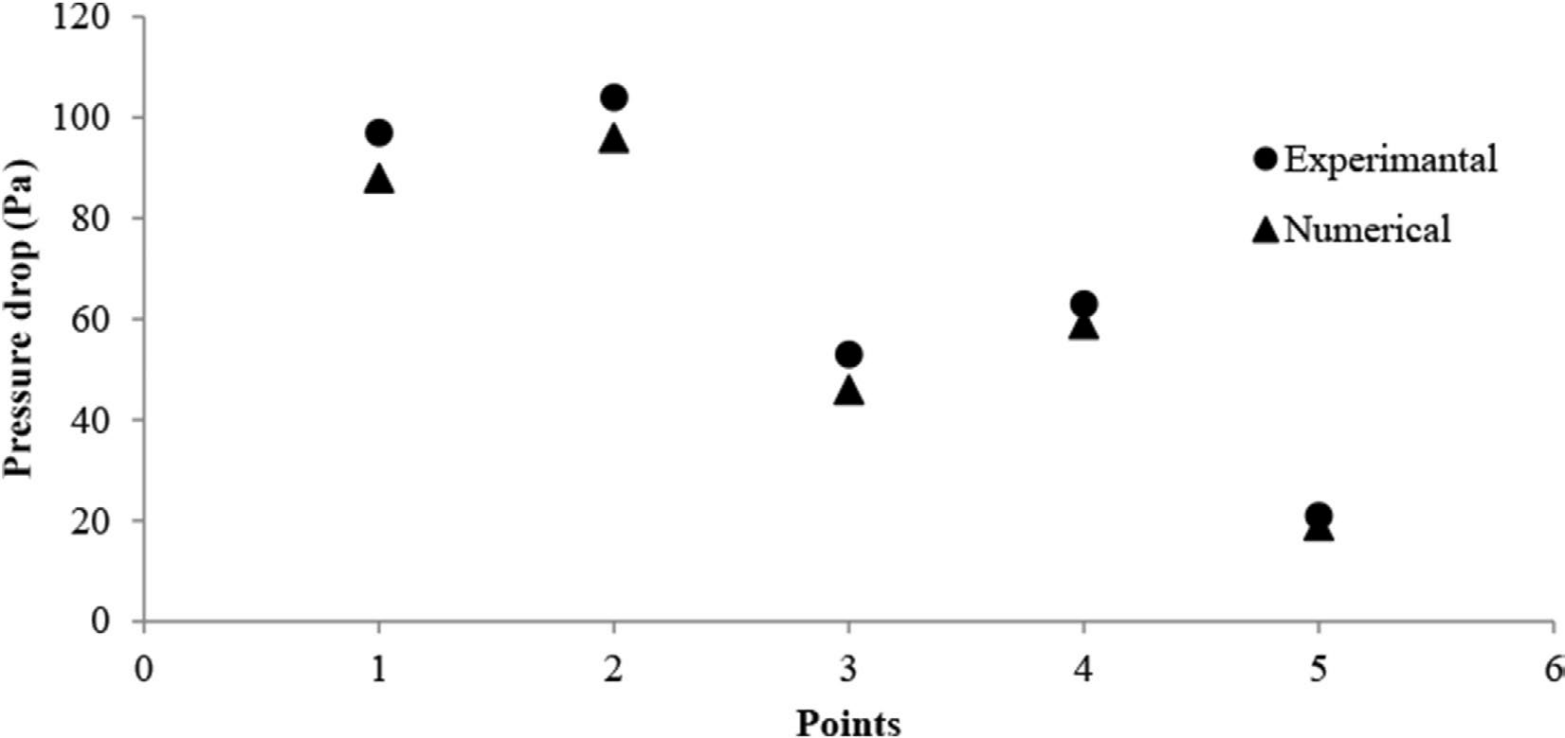
Validation process for pressure drop.

Validation is a crucial step in verifying the reliability of numerical solutions. In the present research, the pressure drop data were employed for this validation process. As illustrated in Figure 6, the validation results demonstrated a strong alignment between the simulation outcomes and the experimental data.

### 3.3 Velocity Magnitude

**FIGURE 7:**
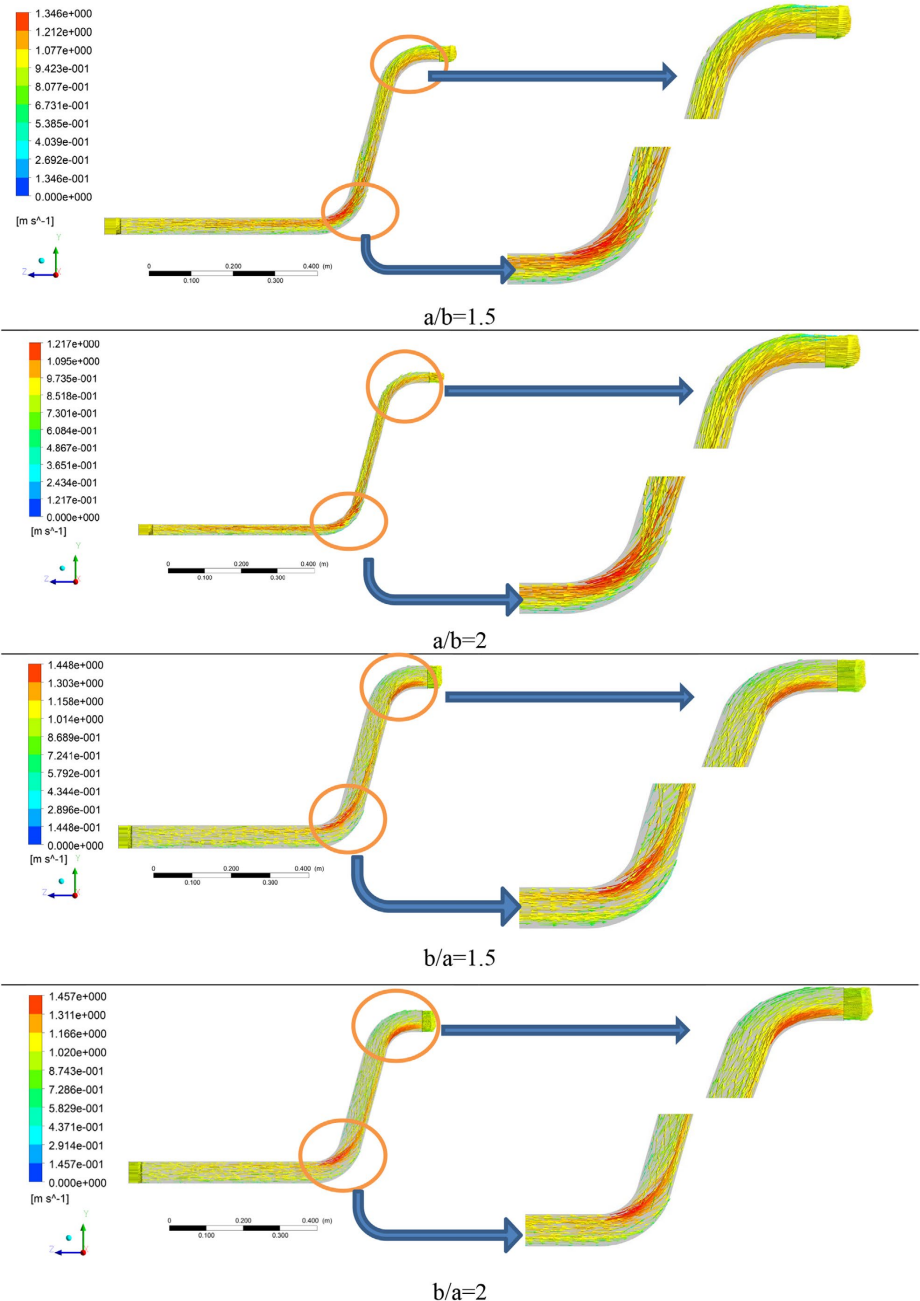
Dimensionless velocity vectors in different pipe cross‐section ratios.

Velocity magnitude can provide a useful understanding of materials flow through the system by indicating different velocity values. Based on the velocity vector for the cross‐section ratio of a/b = 1.5, presented in Figure 7, the maximum velocity is achieved near the inner diameter of the elbows at a dimensionless value (*V*_*m*_/*V*_in_) of 1.45, where *V*_*m*_ and *V*_in_ are the magnitude and inlet velocities, respectively. Considering Bernoulli's equation, this can be considered the main factor of the high dynamic pressure in these areas. As the elbows approach, the uniformity of the velocity value decreases, and a decreasing velocity gradient is created relative to the inner radius of the elbows, which is due to changes in the flow geometry and the deviation of the fluid flow direction. The variation of velocity toward the outer side of an elbow, where centrifugal force prevails, occurs due to the curvature of the elbow, which generates centrifugal forces that push fluid particles outward, resulting in increased velocity along the outer curve of the bend and is related to the concept of Prandtl's secondary flow (Zahedi and Babaee Rad 2022). Also, the velocity vector for the cross‐section ratio of a/b = 2 is shown in Figure 7, which shows a decreasing trend compared to the cross‐section ratio of a/b = 1.5. In fact, it is due to the dilution of the fluid flow and the wheat grains riding on the air flow. In the mentioned cross‐section ratio, the velocity changes begin from the upper part of the pipe and reach their maximum value at the first elbow.

By changing the direction of the pipe stretching from the horizontal axis to the vertical axis in cross‐section ratio of b/a = 1.5, it is observed that the dimensionless velocity magnitude has reached approximately 1.45, which indicates an increase in the velocity at this cross‐section ratio (b/a = 1.5) compared to the previous two ratios (a/b = 1.5 and a/b = 2). The main cause for this increase in dimensionless velocity magnitude can be considered geometric changes resulting from the reduction in the horizontal dimension of the flow direction and also the reduction in the impact surface of the wheat being transferred on the bottom of the transfer pipe, which finally leads to a drop in the final friction force and better flow of the materials, which is shown as an increase in the dimensionless velocity magnitude.

**FIGURE 8:**
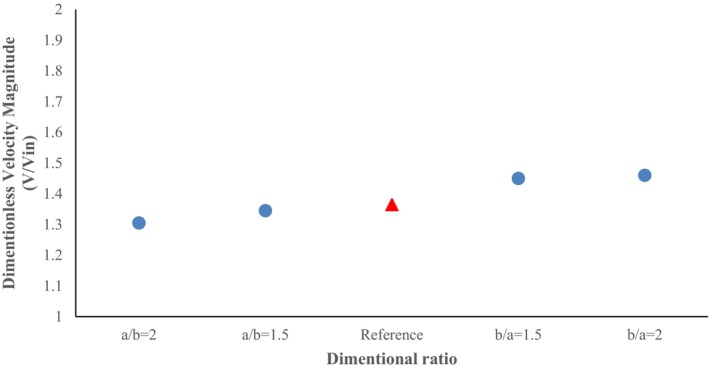
Plot of maximum dimensionless velocity magnitude in different pipe cross‐sections.

**FIGURE 9:**
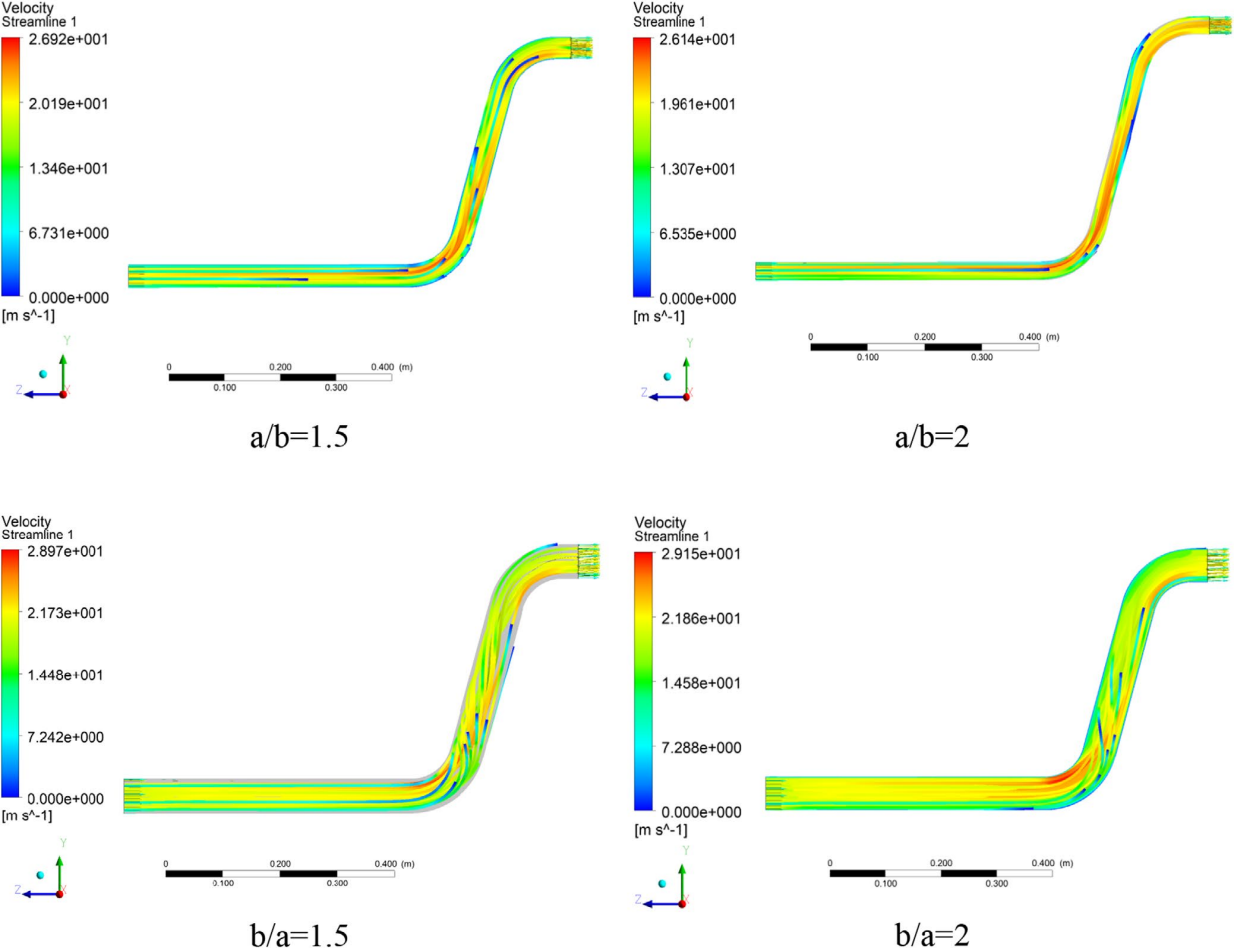
Velocity streamline in different pipe cross‐section ratios.

An important point in the cross‐section ratio of b/a = 2 is the earlier and relatively wider onset of velocity fluctuations in the section before the first elbow, which is achieved due to the more compact horizontal axis of the conveying. Also, a further decrease in the horizontal dimension in this ratio has caused a further increase in the dimensionless velocity magnitude in this cross‐section ratio (b/a = 2), which is the maximum value (0.815) among the 4 cases examined. In order to better compare the dimensionless velocity magnitude in the 4 cross‐section ratios considered, a plot of the maximum changes in the dimensionless velocity magnitude values in different cross‐section ratios is provided in Figure 8. The red triangle‐shaped point in all plots indicates the value related to the base geometry (a/b = 1). In a similar study to the present work, results indicated the presence of regions with high velocity adjacent to the lower section (Moujaes and Deshmukh 2006). Also, streamline plots are presented in Figure 9 to visualize secondary flows, particularly to highlight curvature effects.

### 3.4 Pressure Field

According to conveying solids like wheat through pipes and, in particular, pipes, the analysis of the pressure plays a crucial role. The total pressure is composed of two primary components: static and dynamic pressures. These two pressures are interconnected by the Bernoulli equation, which defines the relationship between static pressure and pressure drop, as well as density and velocity in relation to dynamic pressure.

#### 3.4.1 Static Pressure

**FIGURE 10:**
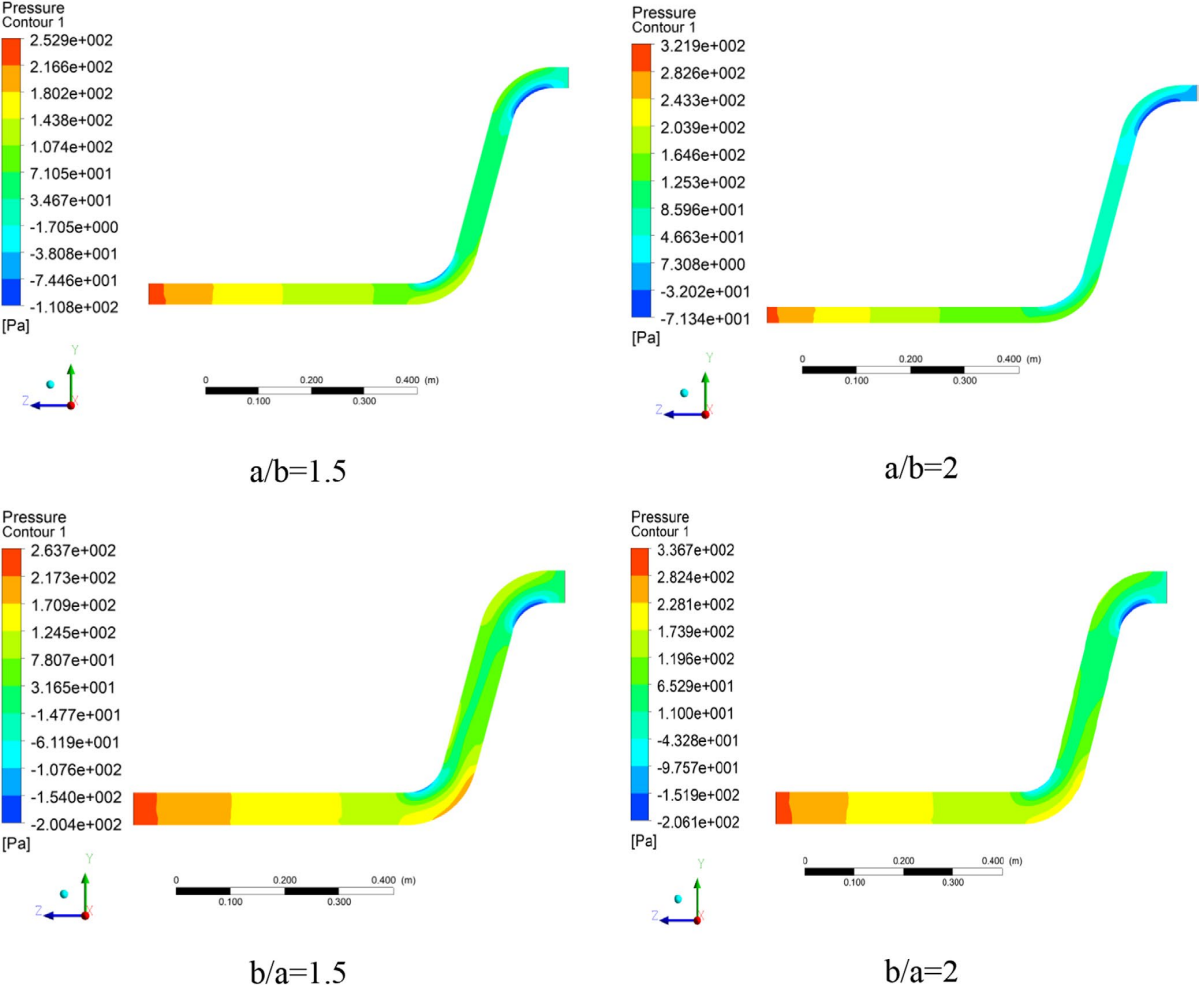
Static pressure contours in different pipe cross‐section ratios.

The static pressure contours in various cross‐section ratios are presented in Figure 10. As shown in a/b = 1.5 ratio, the static pressure after the inlet shows a decreasing behavior in the pipe direction, and an increasing‐decreasing value is observed when reaching the first elbow. The increase value is assigned to the outer radius of the elbow, and the decrease value is assigned to its inner radius. The reason for this increasing‐decreasing behavior in the elbow can be justified according to Bernoulli's equation. Due to the constant value of the total (stagnation) pressure, the static pressure is low at the inner radius due to the higher velocity, and by the same reasoning, the static pressure is relatively high at the elbow due to the lower velocity.

**FIGURE 11:**
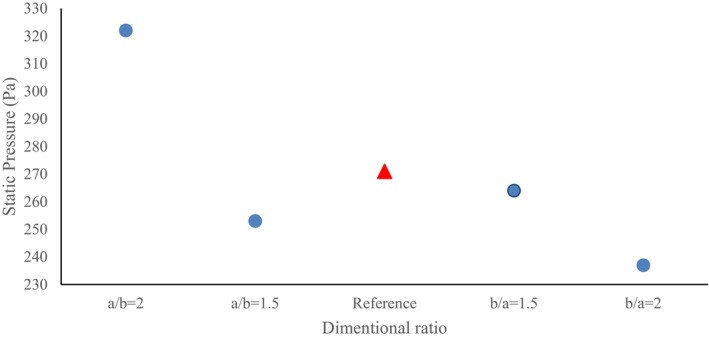
Plot of maximum static pressure in different pipe cross‐section ratios.

After passing the first elbow, the static pressure value remains relatively constant while climbing the inner wall of the pipe, and after reaching the second elbow, a similar trend to the first elbow is observed, and then the fluid flow exits the conveying pipe. The highest static pressure value at this cross‐section ratio is 252.90 Pa. The static pressure value in the first elbow is 135 Pa, and in the second elbow is 99 Pa (Figure 11). Moreover, a negative static pressure is achieved in the inner part of the elbows, with the maximum value belonging to the second elbow. The main cause of this negative pressure is the geometric features of the conveying pipe at the elbows.

Similarly, the pressure contour exhibited significant variations, transitioning from the highest values along the outer radius to minimum values at the inner radius (Moujaes and Deshmukh 2006). Furthermore, in another study about elbows, pressure contours demonstrated a notable trend of enhancement and reduction as air velocity increased from 15.23 m/s^−1^ to 45.72 m/s^−1^ (Mazumder 2012).

#### 3.4.2 Dynamic Pressure

**FIGURE 12:**
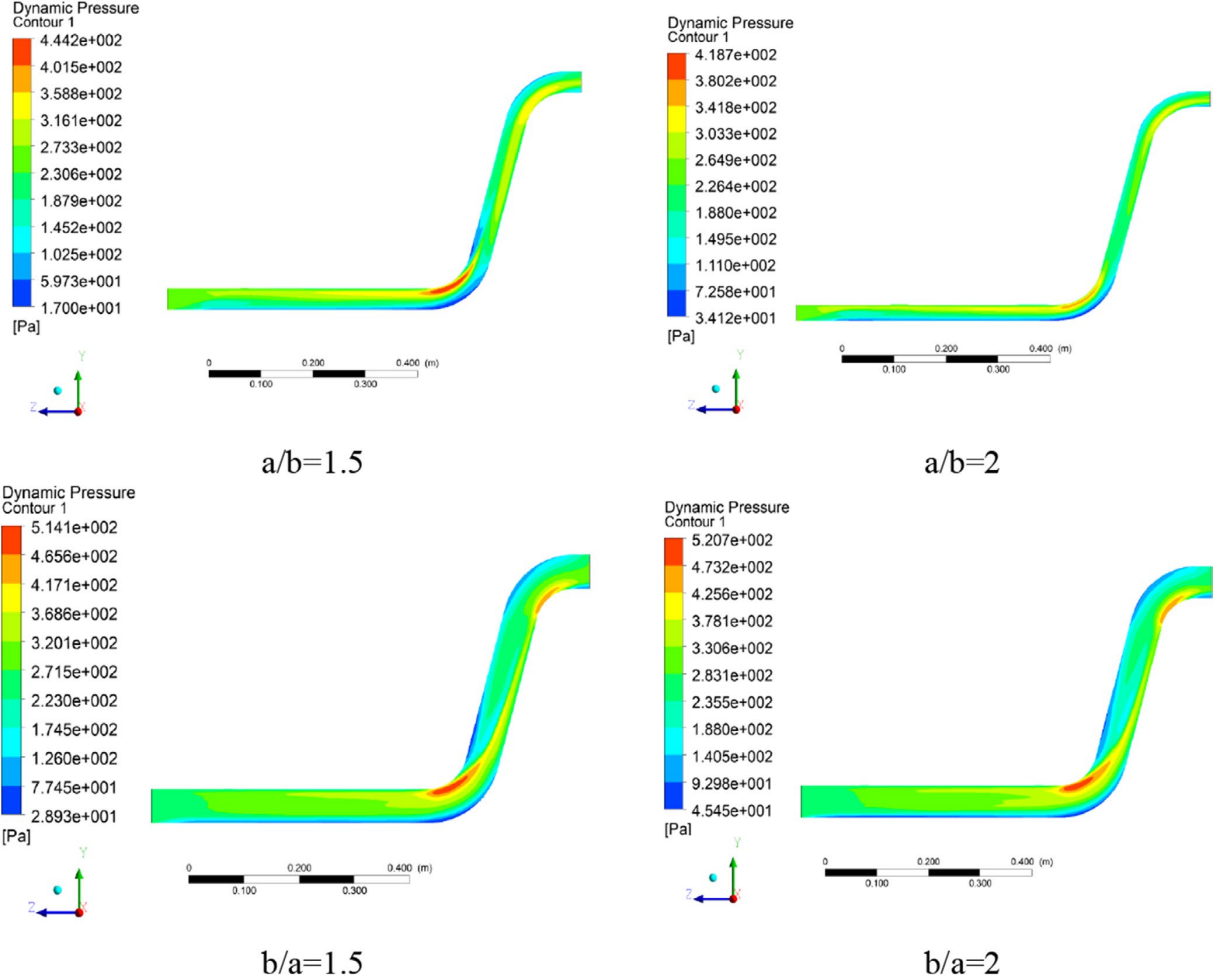
Dynamic pressure contours in different pipe cross‐section ratios.

Figure 12 shows the dynamic pressure contours for all cross‐section ratios. The maximum dynamic pressure at *a/b* = 1.5 cross‐section ratio is 2.444 Pa, which is created at the radius of the first elbow, and considering the relationship between dynamic pressure and velocity and incompressibility (Mach number below 0.3), it has a similar trend to the velocity vector. High dynamic pressure values in the inner sections of the pipe indicate that the fluid flow is transferred in the dilute phase and indicate that no flow blockage has occurred.

At the cross‐section ratio of a/b = 2, there is a high gradient in terms of dynamic pressure along the inner radius of the first elbow relative to its outer radius, which is due to high velocity changes caused by the geometric change and the lengthening of the horizontal axis of the conveying pipe. Also, at this cross‐section ratio, due to the movement of the fluid flow with relatively high dynamic pressure values, the flow has not entered the dense phase, and the conveying has occurred in the dilute phase. It can be concluded that lengthening the cross‐section of the fluid flow pipe (changing from the cross‐section ratio of a/b = 1.5 to a/b = 2) has caused a decrease in the maximum dynamic pressure values. For the cross‐section ratio b/a = 1.5, by changing the cross‐section ratio of the fluid flow conveying pipe in the vertical direction, the contour pattern related to the dynamic pressure has experienced sharp changes.

**FIGURE 13:**
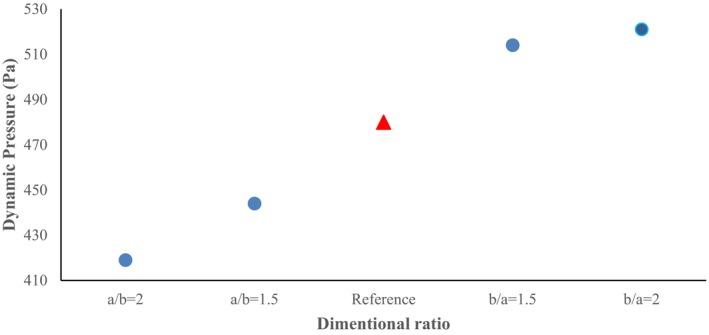
Plot of maximum dynamic pressure in different pipe cross‐section ratios.

Unlike the previous two cases (a/b = 1.5 and a/b = 2), high dynamic pressure values have also been created relatively in the second elbow. Also, in addition to the outer radius of the first elbow, the two areas after the inner radius of the first and the outer radius of the second elbows (the dark blue sections) have relatively low dynamic pressure values, which means relatively low fluid flow velocity in these areas. The maximum dynamic value at this cross‐section ratio is 514.1 Pa (Figure 13). Finally, at the cross‐section ratio of b/a = 2, the static pressure shows a similar trend compared to the cross‐section ratio of b/a = 1.5. As a result, the vertical elongation of the pipe has caused a small enhancement in the dynamic pressure value from 514.1 to 520.7 Pa.

### 3.5 Erosion Rate

**FIGURE 14:**
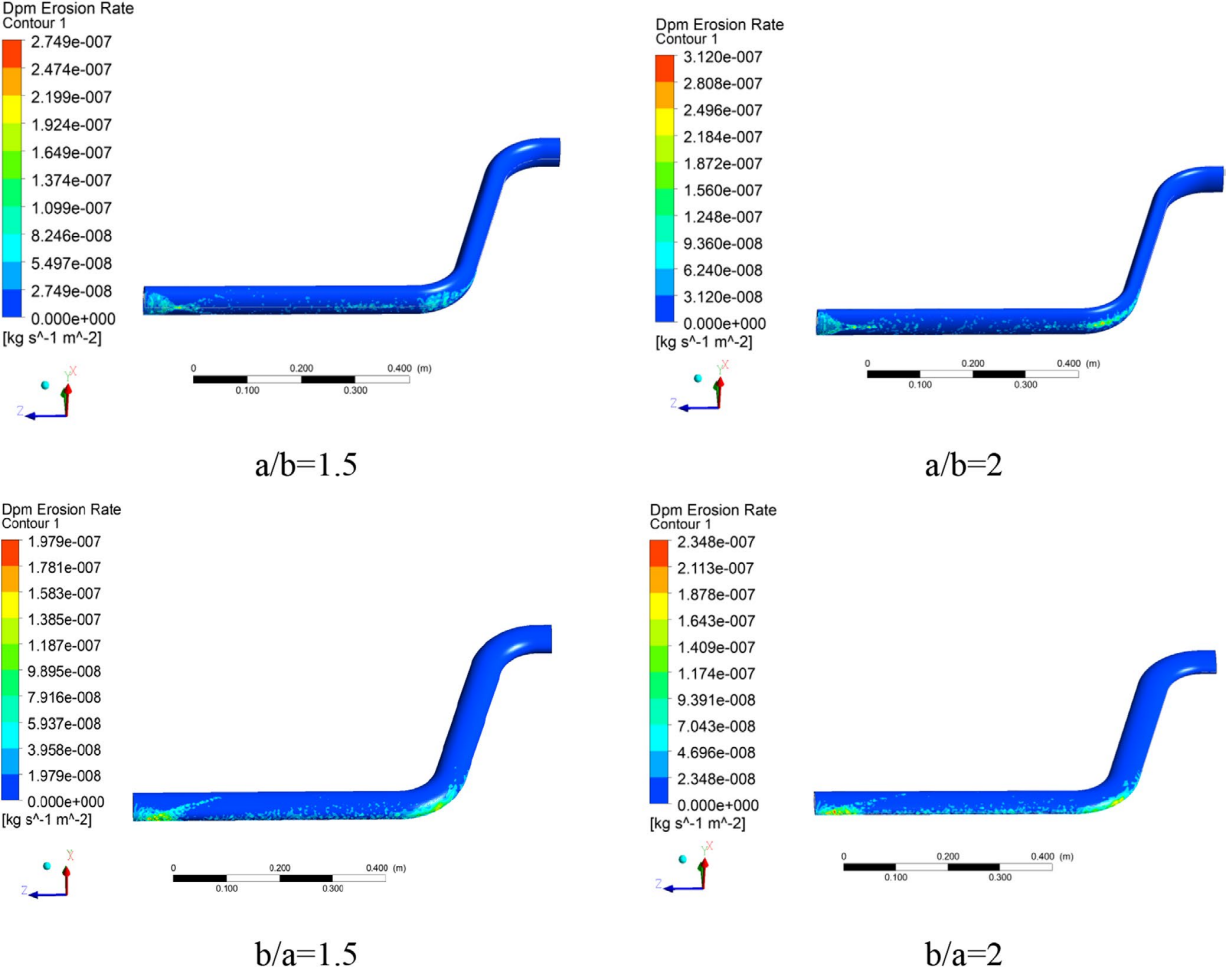
Erosion rate contours in different pipe cross‐section ratios: Bottom view.

**FIGURE 15:**
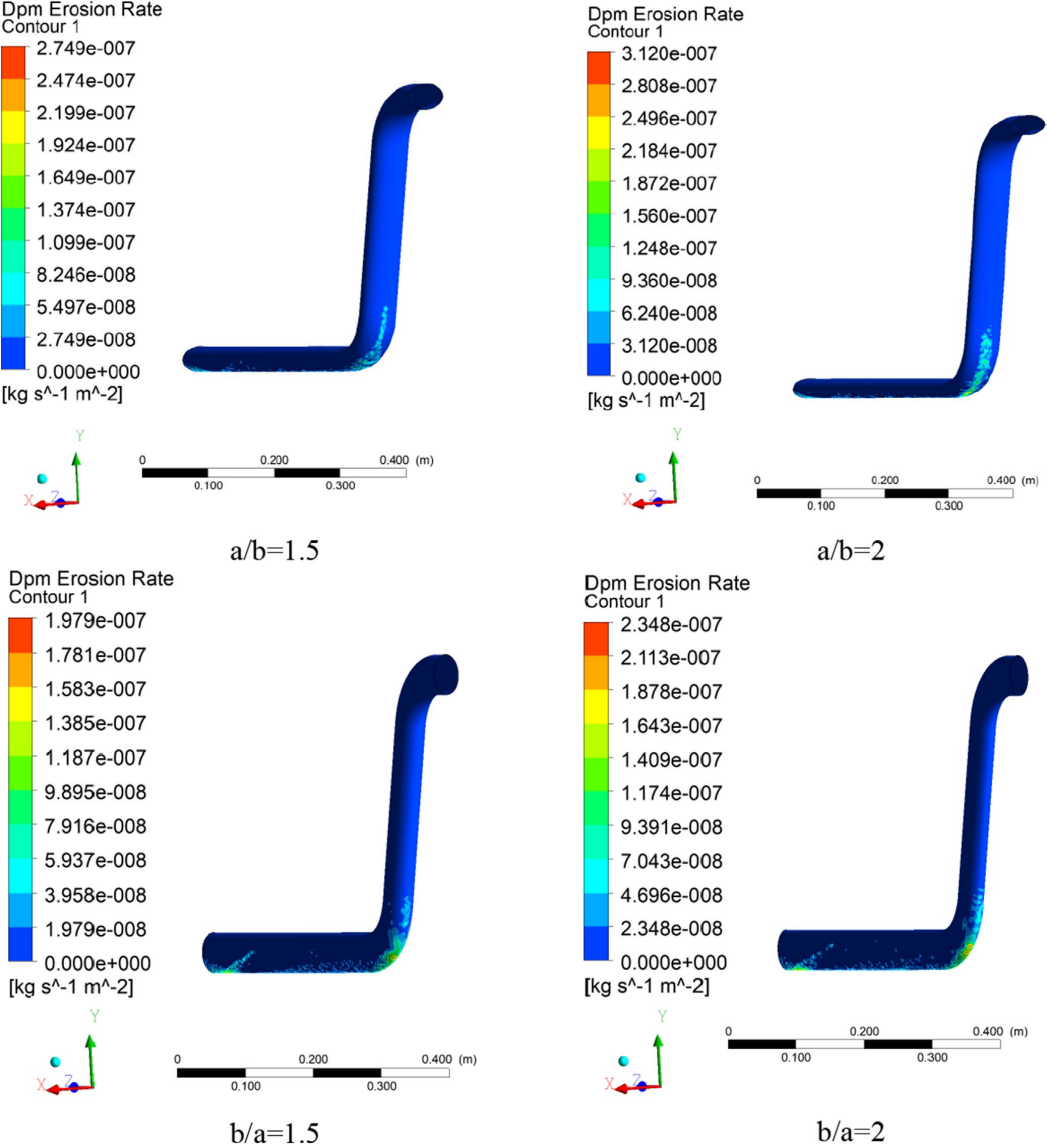
Erosion rate contours in different pipe cross‐section ratios: Side view.

The erosion rate is one of the most critical factors in material conveying inside the pipes. Since material transport mainly involves the collision of particles and their movement on the surface, frictional erosion is inevitable in these processes. Considering the high importance of the erosion phenomenon in the pipe walls, and in order to better observe this phenomenon, three views of the relevant contour, including a downward view, an upward view, and a side view, are presented. Figures 14 and 15 show the erosion rate contours for different cross‐section ratios from the upward and side views, respectively.

Due to the longer horizontal cross‐section of the pipe in a/b = 1.5 ratio and the possibility of more particles colliding with the pipe wall at the beginning of the fluid flow tube, relatively high values of the erosion rate have been obtained, which have gradually decreased due to the wheat grains riding on the carrier fluid. Also, the erosion rate in the second elbow is low, which is achieved due to the rapid flow of the fluid in the outlet section and the designed geometry. The maximum erosion rate at the cross‐section ratio of a/b = 1.5 is 2.75 × 10^−7^.

Moreover, the maximum erosion rate at the cross‐section ratio of a/b = 2 has increased and reached 3.12 × 10^−7^, which shows an increase of 13.45%. This is due to the contact surface of the wheat particles and dimensional changes (the horizontal section of the transmission pipe has become longer). Also, the surface involved in the erosion has become wider than in the previous scenario and shows a higher value (red sections) in terms of concentration in the first elbow, which is clearly visible in the contour of the side view of the erosion rate.

Due to the geometric changes in the cross‐section ratio of b/a = 1.5, the focus of the erosion rate is transferred to the inlet section and erosion at the beginning of the conveying pipe. Actually, this geometric condition reduces the concentration of erosion in the elbow. The maximum erosion rate in this cross‐section ratio (b/a = 1.5) is 1.98 × 10^−7^.

**FIGURE 16:**
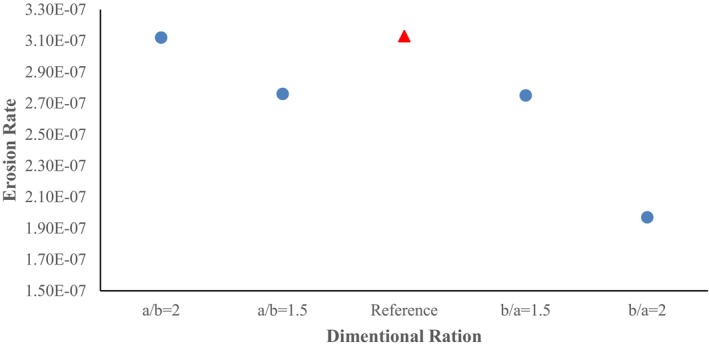
Plot of maximum erosion rate in different pipe cross‐section ratios.

Considering the momentum of the flow and the geometry in the second elbow, the erosion rate in this elbow is not significant. In the cross‐section ratio of b/a = 2, the maximum erosion shows a relatively high increase (18.68%) compared to the cross‐section ratio of b/a = 1.5. The main difference of this cross‐section ratio compared to other cross‐section ratios is the creation of the high erosion rate in the initial elbow (red sections), which is well shown in Figure 16.

The use of twisted pipes has shown the potential to reduce erosion by about 33% (Duarte and de Souza 2017). Additionally, a combined approach involving the distribution function and a flow model was introduced. This method offers a considerably faster alternative to more complex techniques like CFD‐DEM (Uzi et al. 2017).

### 3.6 Vorticity Magnitude

**FIGURE 17:**
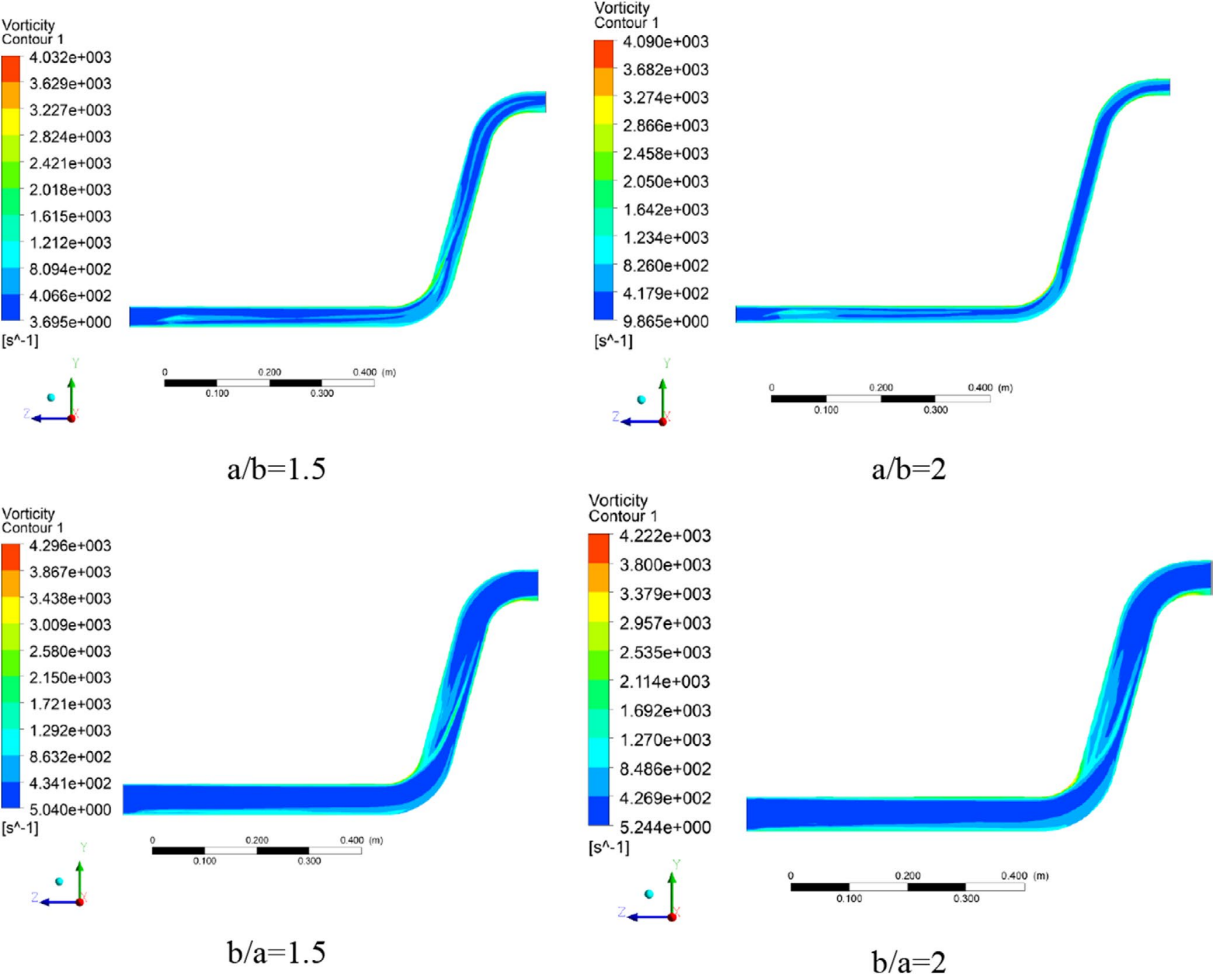
Vorticity magnitude contours in different pipe cross‐section ratios.

The vorticity magnitude contour in conveying materials is momentous for a better understanding of possible blockages and rotation locations. The vorticity magnitude contours for different cross‐section ratios are shown in Figure 17. In sections where the vorticity magnitude is high, the fluid flow movement is challenged and blocked due to the created rotational flows in a/b = 1.5. Due to the change in flow direction in the elbows, the focus on creating vorticity in these areas is clearly visible, and after the first elbow, the maximum vorticity value occurs. It can also be seen how the flow has created vorticity after passing through the elbow and near the wall. The investigation of the vorticity contour shows that its value is mostly in the inner radius of the first elbow and also in the second elbow, and finally before the pipe outlet by the magnitude of 4.03 × 10^3^.

Also, the maximum vorticity magnitude in a/b = 2 cross‐section ratio has remained almost constant compared to the previous scenario, but the relative increase in the vorticity size in the horizontal section of the pipe and its significant decrease in the financial pipe have been achieved due to the geometric changes. Also, the inner radius of the two elbows in the wheat conveying pipe is a potential location from the point of view of creating vorticity.

**FIGURE 18:**
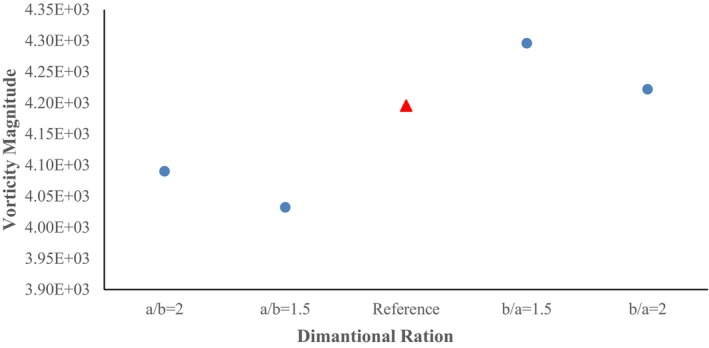
Plot of maximum vorticity magnitude in different pipe cross‐section ratios.

In the cross‐section ratio of b/a = 1.5, in addition to the increase in the vorticity magnitude, major changes in the patterns of vorticity are created in the wheat conveying pipe. Especially in the horizontal section of the pipe, where, except for the bottom wall of the pipe, the vorticity magnitude has decreased noticeably. In contrast, the concentration of vorticity created after the first elbow can be problematic from the point of view of wheat conveying in the dilute phase and cause transferring to dense phases. The maximum value of the vorticity magnitude at the cross‐section ratio of b/a = 2 shows a slight decrease compared to the previous case and has reached a value of 4.22 × 10^3^ (Figure 18). Except for limited cases such as a limited increase in vorticity at the inner and outer radius of the first elbow, no noticeable change is observed compared to the previous ratio (b/a = 1.5), and the created pattern is almost similar to the previous case. Actually, high vorticity regions, like those found in turbulence, are areas where energy is lost due to friction and viscous dissipation. This dissipation drives the mixing process, breaking down larger structures into smaller eddies and ultimately distributing energy. Strong vorticity can also contribute to erosion by creating shear stresses and eddies that erode materials, especially in areas like hydraulic jumps or where there is significant air entrainment.

In a numerical analysis, the authors reported the formation of a reversal flow within a 90° elbow. They also observed that the pressure drop was minimal in the direct part (Moujaes and Deshmukh 2006).

### 3.7 Turbulence Intensity

**FIGURE 19:**
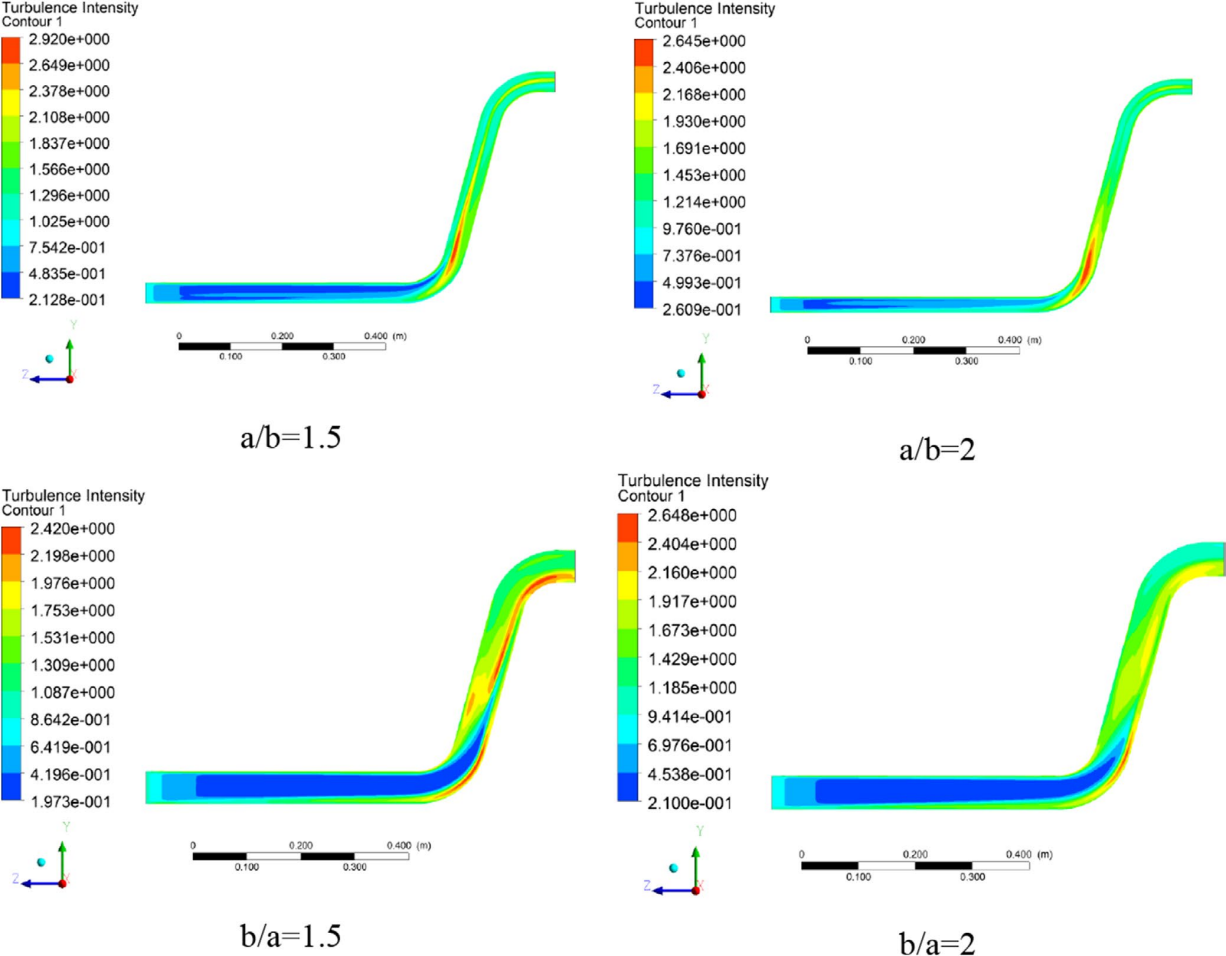
Turbulent intensity contours in different pipe cross‐section ratios.

The turbulence intensity is defined as the ratio of the velocity fluctuations divided by the average velocity. According to Figure 19, the maximum turbulence intensity value belongs to the region after the first elbow. The horizontal section of the transfer pipe has relatively low values of turbulence intensity. In comparison to the previous case, the turbulence intensity has decreased by 9.5% and has reached 2.64. This reduction can be useful in terms of velocity fluctuations and vibrations caused by successive changes in velocity. Also, after the first elbow, the number of areas with a noticeable decrease in turbulence intensity is visible.

**FIGURE 20:**
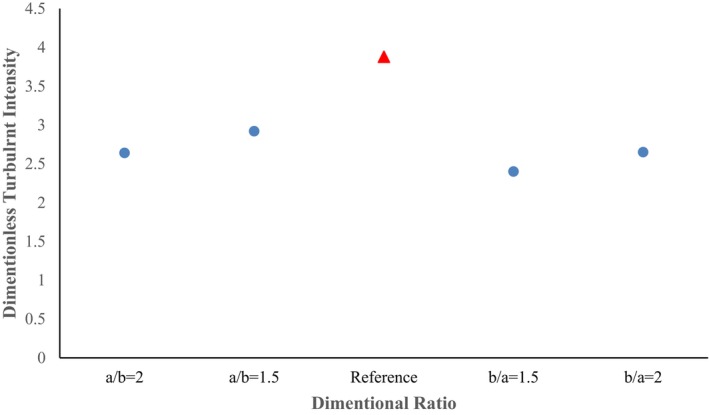
Plot of maximum turbulent intensity in different pipe cross‐section ratios.

In the two cross‐section ratios of b/a = 1.5 and b/a = 2, in terms of turbulence intensity, a decreasing‐increasing behavior is observed, where the turbulence intensity value increased to 2.65 after decreasing to 2.42 (Figure 20). Despite the decrease in the maximum turbulence intensity value in the cross‐section ratio, the previous pattern of turbulence intensity has changed significantly and is extended more to the lower part of the pipe wall, which can impose system vibrations on the system due to velocity fluctuations caused by turbulence intensity. Accordingly, in terms of turbulence intensity, among all the scenarios proposed for the cross‐section ratio, the cross‐section ratio of a/b = 1.5 and a/b = 2 presents the best scenarios.

Higher vorticity indicates increased energy dissipation, stronger mixing, and potentially higher erosion rates in zones where these occur. This is because turbulence accelerates the conversion of kinetic energy into heat, creating turbulent eddies and shear forces that can erode materials and mix fluids more effectively. A related study further revealed that maximum turbulence tended to occur along the interior wall of the elbow (Kannojiya et al. 2018).

## 4 Conclusion

In this study, a comprehensive investigation of the effect of inlet cross‐section variations on the behavior of a fluid containing wheat in a larvae killer system was conducted. In addition to experimental data for validation and boundary conditions, numerical results including velocity magnitude, static and dynamic pressures, strain rate, turbulence intensity, and vorticity magnitude were evaluated in all scenarios. The most remarkable findings obtained from the evaluations are as follows:

- A good correlation between experimental and numerical results was achieved by a maximum error of 10% in various points of the conveying pipe.
- Applying DPM and RSM models to model the solid‐air interaction and fluid turbulence was the best selection.
- The inner radius of the elbows, and especially the first elbow, were the areas where the maximum dimensionless velocity magnitude was observed in these sections. The lowest maximum dimensionless velocity magnitude was also obtained at the inlet ratio of a/b = 2.
- In all cross‐section ratios, the static pressure value showed a decreasing behavior after the inlet and after reaching the first elbow; this behavior changed into an increasing‐decreasing gradient along the first elbow radius, with the enhancing direction toward the outer radius of the elbow and the decreasing direction toward its inner radius, which was fully justified by Bernoulli's equation.
- In terms of erosion, the maximum value belonged to the inlet ratio of a/b = 2, which was mainly due to the wider contact area of the inner surface of the fluid flow tube a/b = 2, and the two inlet ratios a/b = 1.5 and b/a = 1.5 did not provide relatively close values for erosion.
- The lowest vorticity magnitude was obtained at the cross‐section ratio of a/b = 1.5, which allows the use of higher velocities to achieve higher efficiency in this scenario.
- In terms of turbulence intensity, a decreasing‐increasing behavior is observed in the two cross‐section ratios of b/a = 1.5 and b/a = 2, which can lead to periodic vibrations in the system and reduce the life cycle of the system.

To sum up, it can be concluded that considering all scenarios among the four cross‐section ratios, the ratio of a/b = 1.5 was proposed as the most appropriate selection.

## Nomenclature

*P*
: Pressure (Pa)
*u*
: Velocity (m.s^−1^)
h: Height (h)
d: Diameter (m)
*C*_*d*_
: Drag coefficient
*g*
: Gravitational acceleration (m.s^−2^)
*S*
: Source term
$H_{v}$
: Vickers number (Gpa)
*D*_*ij*_
: Stress diffusion (Pa)
$p_{\mathrm{ij}}$
: Shear production
∏ij
: Pressure strain term
$\frac{\partial }{\partial t}\left(\rho \;\bar{u_{i}^{\prime }u_{j}^{\prime }}\right)$
: Stress local derivatives
$\frac{\partial }{\partial x_{k}}\left(\rho u_{k}u_{i}^{\prime }u_{j}^{\prime }\right)$
: Convective transport

**Subscripts**
*p*
: Particle
*g*
: Gas
*'*
: Reference condition
m: Material
*i*
: *x* direction
*j*
: *y* direction
*k*
: *z* direction

**Abbreviation**
CFD: computational fluid dynamics
RSM: reynolds stress model
DPM: discrete phase model
ER: erosion rate

**Greek Symbols**
μ
: Dynamic viscosity (Pas.s)
ρ
: Density (kg.m^−3^)

## Author Contributions

**Ali Ebadi:** data curation; formal analysis; visualization; writing original draft preparation. **Adel Rezvanivand Fanaei:** conceptualization; supervision; project administration; writing – review and editing; software. **Ali Hassanpour:** validation; formal analysis; visualization; writing – review and editing. **Vahid Rostampour:** investigation; data curation; resources.

## Ethics Statement

The authors have nothing to report.

## Conflicts of Interest

The authors declare no conflicts of interest.

## Data Availability

The data that support the findings of this study are available from the corresponding author upon reasonable request.
